# Antifungal Packaging Film to Maintain Quality and Control Postharvest Diseases in Strawberries

**DOI:** 10.3390/antibiotics9090618

**Published:** 2020-09-18

**Authors:** Valentina Trinetta, Austin McDaniel, Konstantinos G. Batziakas, Umut Yucel, Londa Nwadike, Eleni Pliakoni

**Affiliations:** 1 Food Science Institute, Kansas State University, Manhattan, KS 66506, USA; mcdanaus@ksu.edu (A.M.); yucel@ksu.edu (U.Y.); 2 Department of Animal Sciences and Industry, Kansas State University, Manhattan, KS 66506, USA; 3 Department of Horticulture and Natural Resources, Kansas State University, Olathe, KS 66061, USA; kbatziakas@ksu.edu (K.G.B.); epliakoni@ksu.edu (E.P.); 4 Department of Extension, Kansas State University, Olathe, KS 66061, USA; lnwadike@ksu.edu

**Keywords:** antifungal packaging, strawberries, solid lipid nanoparticle, cinnamaldehyde, pullulan

## Abstract

Strawberries are a highly perishable crop with postharvest losses than reach up to 40%. Cost-effective and sustainable technologies in the form of active packing films can provide a solution. Antimicrobial packaging films were produced from pullulan polymer and Solid Lipid Nanoparticles (SLN) containing 1% w/w cinnamaldehyde. Strawberries were stored at 3 °C for 10 days and 12 °C for 6 days. Microbial and physical quality parameters were evaluated during storage. A reduction of approximately 2 Log CFU/g in yeast and mold population was observed for treated strawberries stored at 3 °C as compared to the control (*p* < 0.05). Yeast and molds counts were significantly lower on day 2 and 4 at 12 °C for treated samples. Strawberries packaged with the active films demonstrated lower respiration rates and the retention of bright red color at both storage temperatures. Active pullulan films were effective in maintaining the desired strawberry quality and reducing fungal decay during refrigerated storage.

## 1. Introduction

Fresh strawberries (*Fragaria × ananassa* Duch.) represent a USD 2.2 billion industry in the United States of America (USA) [1]. Strawberries are highly nutritious, but at the same time extremely perishable due to their susceptibility to deterioration, mechanical injury, postharvest physiological disorders and fungal decay [2]. Therefore, these fruits are considered a delicate commodity with a short shelf-life: losses during post-harvest storage are estimated to be as high as 40% [3]. Rapid cooling after harvest and storage at 0–4 °C is the most effective method for maintaining strawberry quality [4]. However, assuring proper storage and preventing temperature abuse during the transportation chain is not always possible [5]. Moreover, carefully handling strawberries during harvesting, packing and postharvest operations is key. Fruit subjected to mechanical damage is more susceptible to plant pathogen infections, and is thus less attractive for consumers [6].

Fungal pathogens are one of the main causes for strawberries postharvest losses. The physiological characteristics of strawberries, such as low pH, optimum high water activity, high sugar concertation and soft texture, provide an ideal environment for fungal growth and infection [2]. The most common fungal pathogens affecting strawberries are *Botrytis cinerea*, *Rhizopus stolonifer* and *Penicillium* spp. [7]. *B. cinerea* is a grey mold disease (or *Botrytis* fruit rot) that affects a wide variety of fruits and vegetables. This pathogen can infect strawberries flowers but its effect is more severe to the fruit during growth in the field and particularly after harvest [8]. Grey mold infestation has significant economic impact, since it is the most frequent reason for strawberry rejection at retail levels [9]. *R. stolonifer* is a fast-growing fungus that typically infects fruit after harvest [10]. It is responsible for the disease soft rot (or *Rhizopus* rot) which develops on the fruit surface as thin, fluffy cotton-like structure. Sporulation then forms a dark mass that can cover the entire fruit [11]. *Penicillium* spp., mainly *Penicillium expansum*, causes circular blue and white mold spots on fruits [11].

Synthetic fungicides have been effective in controlling pathogens such as *B. cinerea* [9]. However, their use has been linked to increased pathogen resistance [12] and effects on human health and the environment [4]. At the same time, a growing number of consumers are seeking fresh, natural, chemical-free and high-quality produce [13]. Therefore, there is a need for interventions that address these preferences and at the same time reduce strawberries postharvest losses by assuring safety and maintaining quality during storage and transportation. The increasing demand for bio-based, disposable and biodegradable materials provides the food industry with the opportunity to invest in alternative solutions for food packaging. Among the available biopolymers, pullulan demonstrated excellent high film formability, considerable mechanical strength, flexibility and unique antimicrobial activity [14]. Our laboratory has been working on pullulan packaging film mechanical and antimicrobial properties characterization. We evaluated the mechanical and physical properties of pullulan packaging films loaded with essential oil (EO) nanoemulsions, their antifungal activity against postharvest diseases [15], and the kinetic release of active compounds from these films [16]. We tested several combinations and concentrations of ingredients and active compounds. Our goal was to obtain an alternative material with mechanical and physical characteristics similar to low density polyethylene films (LDPE) and with broad antimicrobial activity. Particularly, the films loaded with cinnamaldehyde Solid Lipid Nanoparticles (SLN) were the most effective in controlling fungal pathogens such as *R. stolonifer* and *Alternaria* spp. in in vitro conditions. Therefore, in the present study, the efficacy of the previously developed pullulan packaging system containing cinnamaldehyde SLN was investigated on the postharvest microbial decay and storage quality of strawberries stored at 3 and 12 °C.

## 2. Materials and Methods

### 2.1. Film Formulation and Packaging Materials

Based on the data collected in our previous studies, pullulan packaging films loaded with 1% cinnamaldehyde sln were selected for this research [15]. Briefly, pullulan (50 g/L), glycerol (5 g/L), xanthan gum (5 g/L), and locust bean gum (5 g/L) were directly added to SLN emulsions (1000 mL) containing 1% cinnamaldehyde. This mixture was stirred for 5 min at 90 °C to obtain final film solutions. Films were then cast by being poured onto an ultraviolet light-sterilized aluminum sheet pan (43 × 28 cm) and allowed to dry for 24 h at room temperature and 40% relative humidity. In order to trigger antimicrobial activity, films were activated by storing them at freezing temperatures (−20 °C) overnight to allow the complete crystallization of SLN and brought back to room temperature where they remain solid. This step allowed for antimicrobial efficacy enhancement as reported in previous study [15]. Cinnamaldehyde concentration in the final films was verified by GC/MS analysis in our previous study [16]. SLN were prepared using a hot-homogenization technique as described by Trinetta et al., 2017 [17]. On the day of experiment, strawberries were packaged into a molded fiber basket (Pactiv corporations, Lake Forest, IL, USA). The control group had no active packaging layer at the bottom of the basket. The treatment group had an active pullulan film to entirely cover the bottom of the container (dimension of 7 × 8 cm), as absorbent pads in berries boxes to protect from bruising, damages and aid the long-term freshness.

### 2.2. Plant Material and Storage Conditions

On the day of experiment, strawberries (*Fragaria ananassa* Duch.) were harvested (May and June 2019) from a local farm located in Edgerton (KS) at commercial ripeness. Fruits were harvested by trained workers and transported within 2 h, at refrigerated conditions, to the Kansas State Urban Food Production and Postharvest Handling laboratory (Olathe, KS, USA). Upon arrival, strawberries were randomly assigned to either the control or the treatment group. Each sample unit included 10 berries for every sampling day. Samples were stored in environmental chambers (ThermoFisher Scientific Inc., Asheville, NC, USA) at 3 or 12 °C with 95% relative humidity. These two temperatures were chosen based on produce growers common practices: 3 °C is the recommended refrigerated storage temperature for crops, while 12 °C is used by small, diversified produce operations in Central U.S. when their cooling capability is limited [18]. This temperature allows storing chilling sensitive and non-sensitive crops together. Microbiological counts, respiration rate, color, firmness, titratable acidity and soluble solids content were evaluated every 2 days, up to 10 days for the samples storage at 3 °C and up to 6 days for the strawberries stored at 12 °C. At each sampling day, a portion of strawberries (20 g) were frozen using liquid nitrogen, stored at −20 °C and used for the later analysis of antioxidant capacity.

### 2.3. Microbiological Analysis

At each sampling day, total aerobic count and yeast and mold count were evaluated. Samples (10 g for each treatment at each day) were aseptically transferred in 90 mL of 0.1% peptone water (Becton, Dickinson and Company, Sparks, MD, USA) and gently hand massaged for 1 min. Appropriate dilutions were plated in duplicate onto Rapid Yeast and Mold (RYM) petrifilm and Rapid Aerobic Count (RAC) petrifilm (3M, Saint Paul, MN, USA). RYM plates were incubated at room temperature (21 ± 2 °C) for 48 h and RAC plates at 37 °C for 18–24 h before enumeration. The experiments were conducted three times per conditions.

### 2.4. Respiration Rate

Strawberry respiration rate was evaluated using the closed system method [19]. At each sampling day, strawberries (10 g for each treatment at each day) were placed in air-tight glass jars (0.75 L Le Parfait, Villeurbanne, France) equipped with a septum for 1 h at the respective storage temperature. The amount of CO_2_ produced was quantified using a portable gas analyzer (Bridge Analyzer; Bedford Heights, OH, USA). Respiration rate was expressed as the rate of CO_2_ production (mg CO_2_/kg-hr). The experiments were conducted three times per conditions.

### 2.5. Fruit Firmness

A texture analyzer (TA-XT.plus, Texture Technologies Corp., Scarsdale, NY, USA) was used to determined strawberries firmness (g) following the protocol described by Caner et al., 2008 [20]. Two measurements were taken for each sample (one strawberry for each treatment at each day) fruit at different locations using an 8-mm puncture probe (TA-58, Texture Technologies Corp., Scarsdale, NY, USA).

### 2.6. Fruit surface Color

A Chroma-Meter (A5 CR-400, Minolta Co. Ltd., Osaka, Japan) was used to determine fruit surface color. Color measurements were performed on the opposite shoulder sides of each sample (one strawberry for each treatment at each day). The results were expressed following the CIELAB color system: L* (-darkness to +lightness) and a* (-greenness to +redness). The experiments were conducted three times per conditions.

### 2.7. Titratable Acidity

Titratable acidity was measured with an automatic titrometer (Compact Titrosampler 862, Metrohm USA Inc. Riverview, FL, USA). At each sampling day, strawberries (10 g for each treatment at each day) were analyzed and the results were expressed as a % of citric acid. The experiments were conducted three times per condition.

### 2.8. Soluble solids Content

Soluble Solid Content (SSC) was measured using an electronic refractometer (Reichert Technologies, Depew, NY, USA) and expressed in °Brix.). At each sampling day, strawberries (10 g for each treatment at each day) were analyzed and the results were expressed as a % of citric acid. The experiments were conducted three times per conditions.

### 2.9. Antioxidant Capacity

For each sampling day and group, previously frozen strawberries (20 g) were homogenized using a mill (IKA Laboratory, Analytical & Processing Equipment, Wilmington, NC, USA) with liquid nitrogen and centrifuged at 17,600× *g* (JA-17, Beckman Coulter, Palo Alto, CA, USA) for 20 min at 4 °C. The precipitate was then used for the antioxidant capacity analysis defined in terms of Oxygen Radical Absorbance Capacity (ORAC) and total phenolic content. ORAC (μM TE 100 g^−1^ FW) was measured using the protocol developed by Prior et al., 2003 [21] and total phenolic content (GAE kg^−1^ FW) according to the method of Singleton & Rossi, 1965 [22]. A 96-well microplate reader (Synergy H1, BioTek Instruments, Inc. Winooski, VT, USA) was used to perform all the readings. Briefly a calibration curve was prepared using aqueous solution of gallic acid mixed with 1.0 N Folin–Ciocalteu reagent and 75 g/L sodium carbonate. The absorbance was measured after 30 min at 760 nm and 25 °C and the total phenolics were expressed as gallic acid equivalents (GAE) in milligrams per gram of extract.

### 2.10. Experimental Design and Statistical Analysis

For each temperature, experiments were conducted three times using strawberries from the same farm harvested over 3 different days. This study implemented a generalized randomized block design, with harvest being the blocking factor. The fixed effects of the linear model were harvest, day, treatment. Interaction between treatment and day were evaluated. Least Square Means (LSmeans) and standard errors were reported for each treatment group. Treatment effects were evaluated against control within a given day based on a 2-sided test for non-zero difference. The statistical analysis was executed using the Statistical Analysis Software (SAS version 9.4; Cary, NC, USA) and utilizing the PROC MIXED with Kenward–Roger set as the denominator degrees of freedom methods option (DDFM=KR).

## 3. Results and Discussion

### 3.1. Microbial Analysis

**Figure 1:**
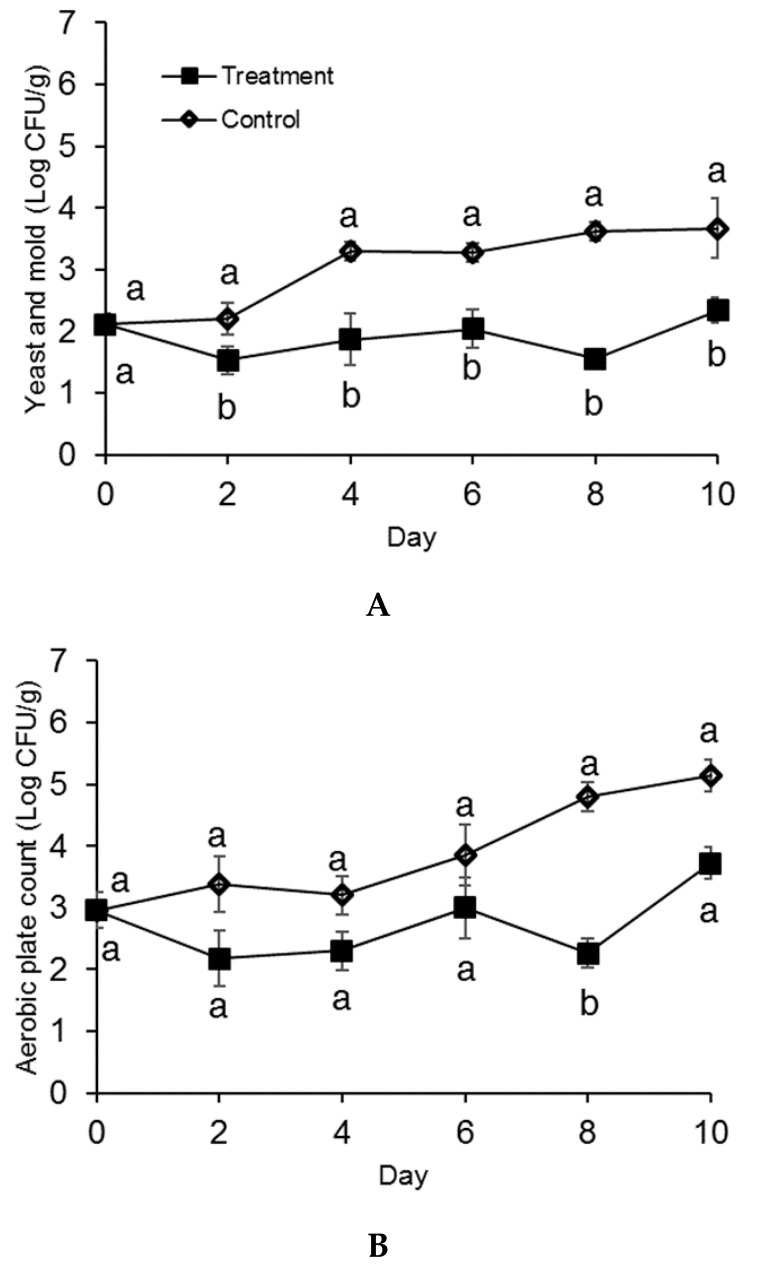
Yeast and mold count (**A**) and total aerobic plate count (**B**) of strawberries stored at 3 °C for 10 days in molded fiber baskets containing pullulan packaging films loaded with 1% *w*/*w* cinnamaldehyde SLN (treatment) or without (control). Different letters (a, b) for each day denotes a significant difference (*p* < 0.05) between the treatment and the control. The error bars represent standard error.

**Figure 2:**
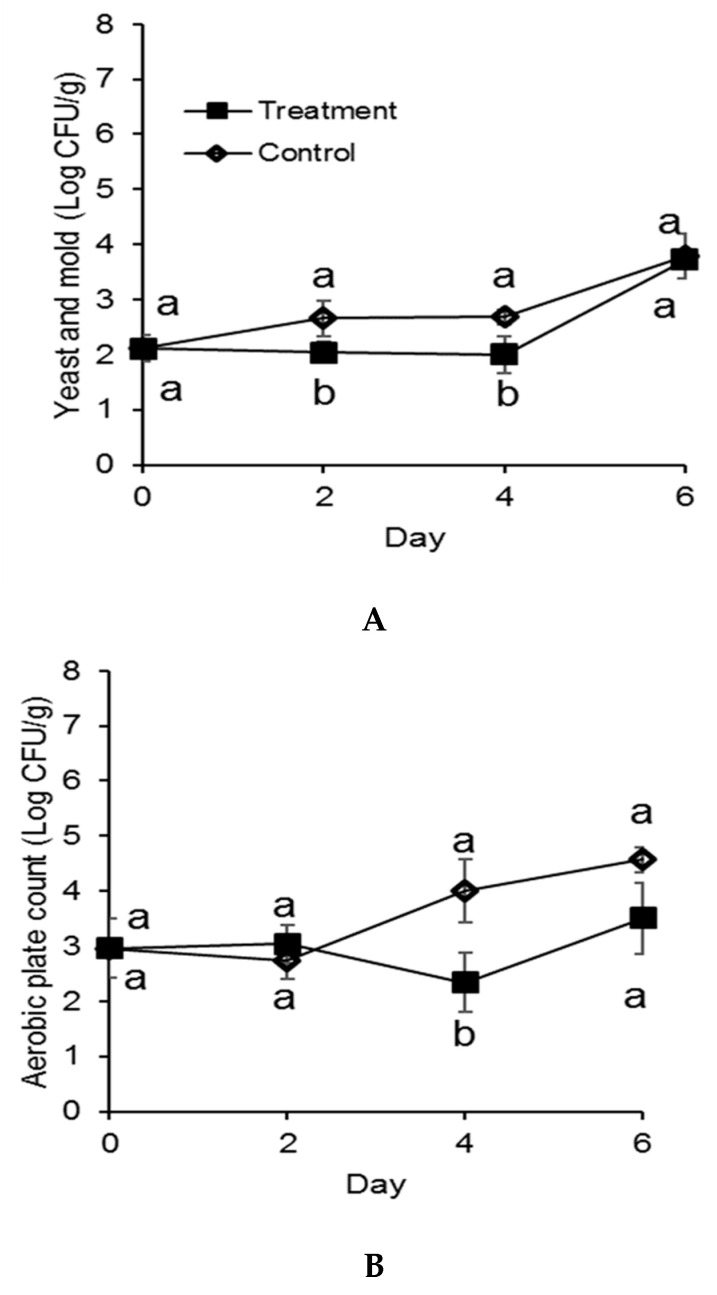
Yeast and mold count (**A**) and total aerobic plate count (**B**) of strawberries stored at 12 °C for 10 days in molded fiber baskets containing pullulan packaging films loaded with 1% w/w cinnamaldehyde SLN (treatment) or without (control). Different letters (a, b) for each day denotes a significant difference (*p* < 0.05) between the treatment and the control. The error bars represent standard error.

Overall, treated strawberries displayed lower yeast and mold counts than the control strawberries at both storage temperatures. Nevertheless, berries packaged in the active system and stored at 3 °C presented a significant lower microbial count only on day 8 (*p* < 0.05), as reported in Figure 1. A maximum reduction of 2 Log CFU/g and 1.5 Log CFU/mL were observed in the treated samples by day 10 in yeast and mold and total plate counts, respectively. An increase in cell count was observed between day 8 and day 10 for treated strawberries, probably due to the fact that the antimicrobial effectiveness of the active system decreases with time. At the storage temperature of 12 °C, a significant difference (*p* < 0.05) between control and treatment was observed for yeast and mold counts only on day 2 and day 4: reductions of 0.7 Log CFU/g and 0.5 Log CFU/mL were observed, respectively. At this storage temperature, strawberries were kept up to 6 days, since they were considered unacceptable for visual quality after 1 week. Overall, there was no significant difference in microbial population between treated and untreated samples, as reported in Figure 2. These results highlight the importance of the correct storage conditions. For strawberries, low storage temperature remains the most effective method for controlling postharvest decay [11]. As demonstrated by Timudo-Torrevilla et al., 2005 [23], the postharvest development of *Rhizopus* spp. can be reduced by temperatures below 6 °C, and storage below 2.5 °C was able to reduce *Botrytis cinerea* [9]. The developed antimicrobial packaging system was more effective in controlling strawberry fungal decay at the optimum storage temperature (3 °C) compared to at the non-optimum temperature (12 °C). Our results highlight a possible positive synergistic effect between the antifungal properties of the active pullulan film and storage temperature. Nevertheless, pullulan packaging films loaded with cinnamaldehyde SLN were not as effective in inhibiting total aerobic bacteria: no significant differences were observed between the treatment and the control for most of the storage life. Only on day 8 for 3 °C (Figure 1B) and day 4 for 12 °C (Figure 2B) where the treatment demonstrated significantly lower (*p* < 0.05) total aerobic plate count: reductions of 1.3 Log CFU/g and 0.7 Log CFU/g were observed, respectively. Similarly, López et al., 2007 [24] reported that polypropylene (PP) and polyethylene/ethylene (PE/EVOH) films incorporated with 4% (*w/w*) cinnamon essential oil were effective in completely inhibiting spoilage fungi growth. However, higher concentrations of active compound (8–10% *w*/*w*) were required to completely inhibit total aerobic growth.

### 3.2. Respiration Rate and Fruit Firmness

**Figure 3:**
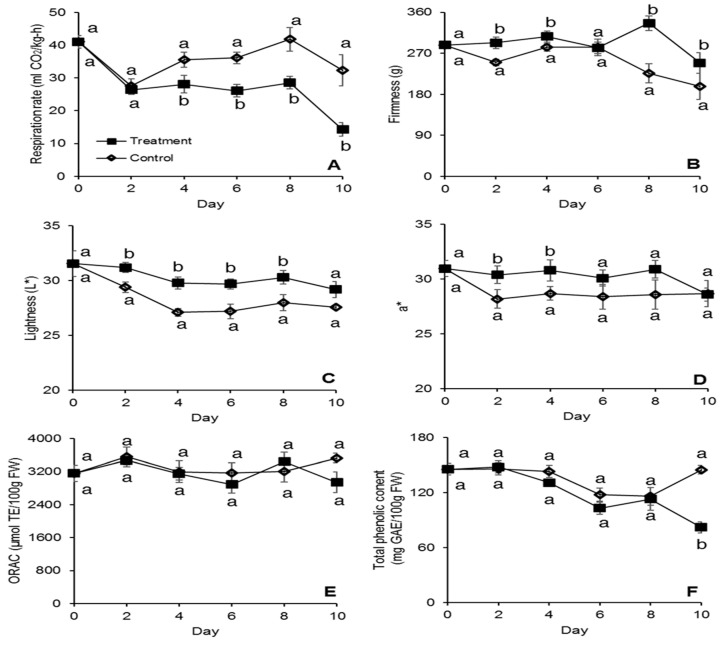
Respiration rate (**A**), fruit firmness (**B**), fruit surface color measured as lightness (L*) (**C**) and a* value (**D**), antioxidant capacity (**E**) and total phenolic content (**F**) of strawberries stored at 3 °C for 10 days in molded fiber baskets containing pullulan packaging films loaded with 1% *w/w* cinnamaldehyde SLN (treatment) or without (control). Different letters (a, b) for each day denotes a significant difference (*p* < 0.05) between the treatment and the control. The error bars represent standard error.

**Figure 4:**
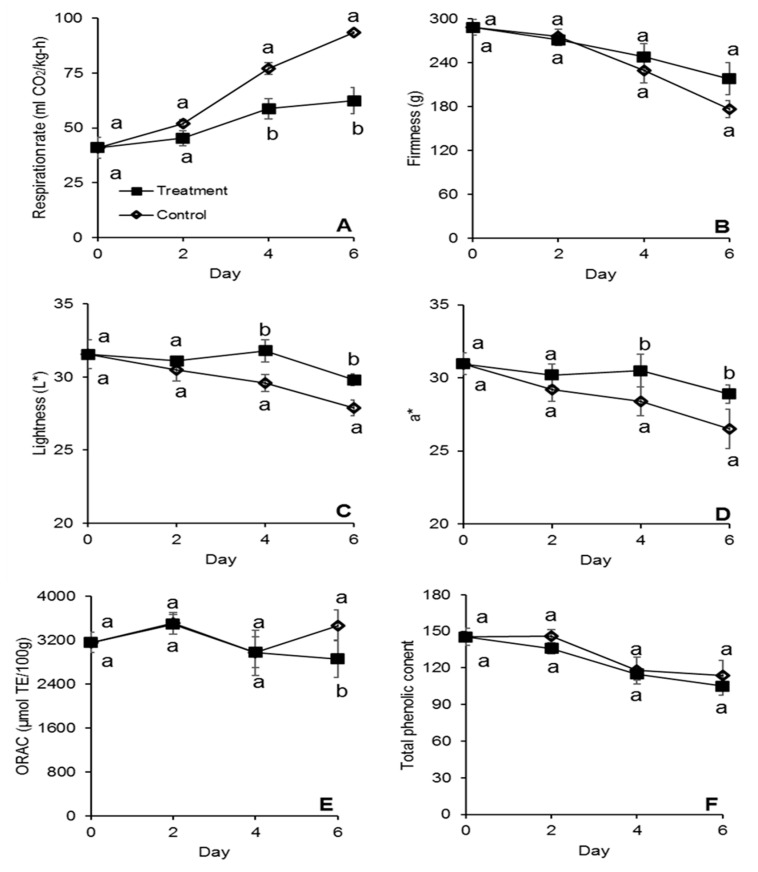
Respiration rate (**A**), fruit firmness (**B**), fruit surface color measured as lightness (L*) (**C**) and a* value (**D**), antioxidant capacity (**E**) and total phenolic content (**F**) of strawberries stored at 12 °C for 6 days in molded fiber baskets containing pullulan packaging films loaded with 1% *w/w* cinnamaldehyde SLN (treatment) or without (control). Different letters (a, b) for each day denotes a significant difference (*p* < 0.05) between the treatment and the control. The error bars represent standard error.

From day 4, Strawberries stored at 3 °C in baskets containing the active packaging layer, demonstrated 1.3 to 2.3 times lower respiration rate (*p* < 0.05) compared to the control (Figure 3A). On the last day of shelf life, a decrease in respiration rate was observed for both treatments (Figure 3A). A similar trend was also for strawberries stored at 4 °C, and this relates with the fruit transitioning to an over-ripe stage. Furthermore, at 12 °C on day 4 and 6, the active packaging reduced the respiration rate 1.3 to 1.5 times (*p* < 0.05) (Figure 4A). The ability of the active film to reduce the respiration rate of the treated fruit is probably related to the decreased fruit damage from fungal decay. It has been reported that fungal growth in strawberries can cause an increase in respiration rate [18,25]. The relationship between respiration rate and shelf-life is inverse [26]: this means that the decrease in respiration rate by the active pullulan film can potentially translate to decreased postharvest losses and a longer shelf life.

### 3.3. Fruit Surface Color, Titratable Acidity and SSC

Loss of firmness is among the most significant changes occurring in strawberries during storage and relates to changes in metabolism and water content [27]. In this study, we observed improved firmness retention (*p* < 0.05) for the treated strawberries stored at 3 °C from day 2 to day 10 of storage, with the exception of day 6, where the fruit firmness was similar between treatment and control (Figure 3B). Sallato et al., 2007 [28] similarly reported that fungicide treatments significantly reduced the fruit softening of strawberries stored at 5 °C. Fungal infections from pathogens such as *Rhizopus* spp. are closely related with the activity of macerating enzymes such as polygalacturonases, xylanase and cellulase, which lead to fruit softening and reduced firmness in strawberries [11]. Thus, the reduction in fungal infections from the use of the active packaging at 3 °C contributed to maintaining fruit firmness. There were no statistical differences in firmness, between control and treatment, for the strawberries stored at 12 °C (Figure 4B). Non-optimum storage temperatures accelerate firmness loss drastically in strawberries [29], which explains why no difference in firmness was observed between the treatment and the control at 12 °C. Fruit firmness is one of the main factors determining strawberry quality and shelf life [27]. Our results indicate that the active packaging system can maintain fruit storage quality and potentially increase shelf life when strawberries are optimally stored at 3 °C.

Moreover, the color of fruits was measured overtime. At 3 °C, the treated strawberries demonstrated significantly less dark surface color (*p* < 0.05) as compared to the control, indicated by the higher L* values, from day 2 to day 8 of storage. No significant difference with the control was instead reported on day 10 (Figure 3C). Moreover, the treated strawberries demonstrated more redness (*p* < 0.05), as indicated by the a* value measured on day 2 and 4 of storage (Figure 3D). At 12 °C, the treated strawberries demonstrated less dark and redder surface color on day 4 and 6 of storage (Figure 4C,D). Hernández-Muñoz et al., 2008 [25] also reported darkening and loss of red coloration in strawberries with increased decay incidence, which was attributed to increased color oxidation. Color is an important quality parameter for consumer purchasing decisions. Particularly for strawberries, the bright red color is one of the main parameters for consumer acceptability [30]. Our results indicate that the active film can maintain the desirable bright red color when strawberries are stored at 3 °C and 12 °C. Notwithstanding, the formulated active packaging technology did not have an effect on the titratable acidity or the SSC of the strawberries stored at 3 °C or 12 °C (Table 1).

### 3.4. Antioxidant Capacity

There were no differences in ORAC values been the treated and untreated strawberries stored at 3 °C (Figure 3E), while the treatment demonstrated significantly lower (*p* < 0.05) phenolic content compared to the control after 10 days of storage (Figure 3F). At 12 °C, the treated strawberries demonstrated significantly lower ORAC values (*p* < 0.05) after 6 days of storage (Figure 4E), while there was no difference in total phenolic content between the treatment and the control throughout storage (Figure 4F). Guerreiro et al., 2015 [31] reported that edible coatings enriched with essential oils had minimal effect on the antioxidant capacity of strawberries during storage, as we observed. The differences in phenolic content on the last day of storage at 3 °C and ORAC on the last day of storage at 12 °C might be attributed to an increased production of antioxidant compounds in untreated strawberries as a response to the biotic stress caused by fungal and bacterial infection [32].

## 4. Conclusions

This study reported the effect of pullulan packaging films loaded with cinnamaldehyde SLN on the postharvest microbial decay and storage quality of strawberries stored in optimum (3 °C) and non-optimum (12 °C) temperatures. The films reduced yeast and mold population at both temperatures, but the effects were more noticeable at 3 °C, as tested in the present study. The active system was not effective in reducing total aerobic counts. Nevertheless, we observed that pullulan packaging films loaded with cinnamaldehyde SLN were instead able to reduce the metabolic rate and maintain the desirable color of strawberries stored at 3 °C and 12 °C. Furthermore, we observed a delayed fruit firmness loss at 3 °C. Our results indicate a positive synergistic activity between optimum refrigerated storage temperature (3 °C) and the effect of the developed active packaging system in terms of extended microbial shelf-life and preserving fruit quality: pullulan films loaded with cinnamaldehyde SLN can increase the shelf life of strawberries stored at 3 °C. Further research is needed to investigate the sensory characteristics and consumer acceptability of treated strawberries.

## Figures and Tables

**Table 1:** Titratable acidity and soluble solid content of strawberries stored at 3 °C for 10 days or 12 °C for 6 days in molded fiber baskets containing pullulan packaging films loaded with 1% *w/w* cinnamaldehyde SLN (treatment) or without (control).

Day	0	2	4	6	8	10
3 °C						
**% Titratable Acidity**						
Treatment	0.65 ± 0.04	0.69 ± 0.04	0.69 ± 0.04	0.70 ± 0.04	0.58 ± 0.05	0.66 ± 0.06
Control	0.65 ± 0.04	0.68 ± 0.03	0.68 ± 0.05	0.68 ± 0.04	0.51 ± 0.06	0.63 ± 0.07
**Soluble Solid Content (°Brix)**						
Treatment	7.33 ± 0.45	7.41 ± 0.68	8.03 ± 0.73	6.57 ± 0.60	6.37 ± 0.58	6.61 ± 0.73
Control	7.33 ± 0.45	7.78 ± 0.82	8.66 ± 0.66	7.13 ± 0.75	5.17 ± 0.62	7.11 ± 0.13
12 °C						
**% Titratable Acidity**						
Treatment	0.65 ± 0.04	0.64 ± 0.04	0.64 ± 0.03	0.50 ± 0.04	-	-
Control	0.65 ± 0.04	0.68 ± 0.05	0.66 ± 0.04	0.54 ± 0.06	-	-
**Soluble Solid Content (°Brix)**						
Treatment	7.33 ± 0.61	6.77 ± 0.62	6.97 ± 0.78	5.91 ± 0.55	-	-
Control	7.33 ± 0.61	7.27 ± 0.49	6.63 ± 0.72	6.73 ± 0.45	-	-
